# Supplementary material for: The SvFUL2 transcription factor is required for inflorescence determinacy and timely flowering in Setaria viridis
Source: Plant Physiol. 2021 Apr 19;187(3):1202–20. doi: 10.1093/plphys/kiab169 (PMC8566296; doi:10.1093/plphys/kiab169)
Supplement: kiab169_Supplementary_Data [file pp.01532.2020-s01.pdf › pp.01532.2020-s01.pdf]

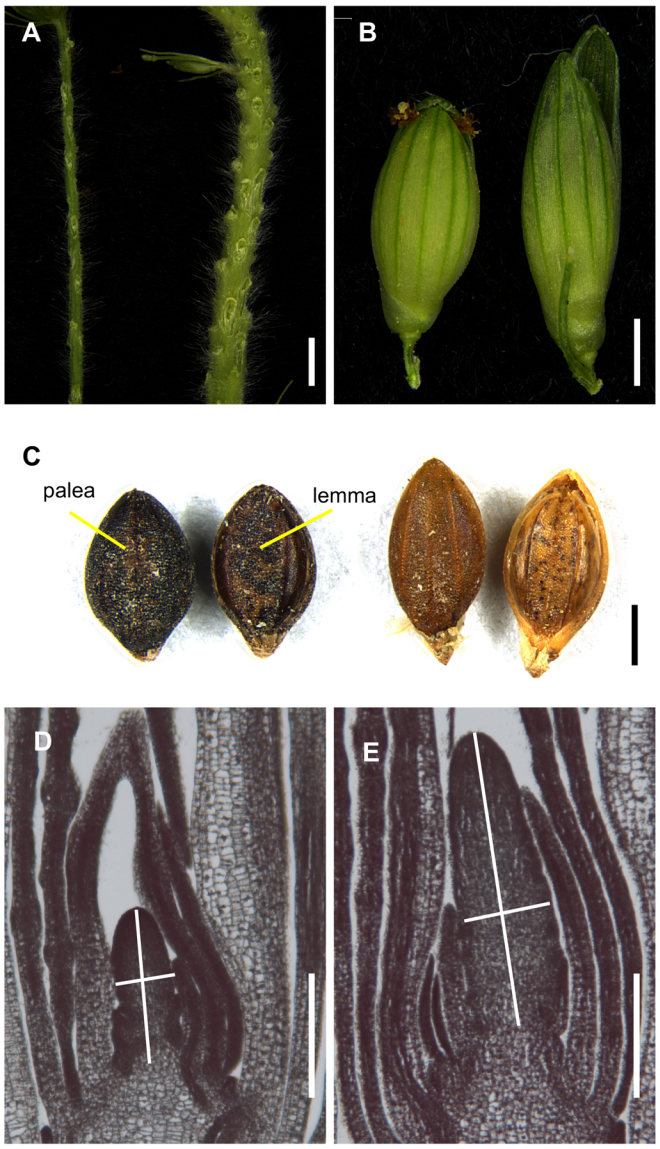

Supplemental Figure S1. Additional characteristics of the *brl1* mutants (Supports Figure 1).

Compared to the wild-type (**left**), *brl1* mutant (**right**) panicles had much thicker panicle axes (**A**) and developed longer spikelets (**B**) with longer lemmas and paleae (**C**). Scale bars = 1mm in **A** and 500μm in **B** and **C**.

At the end of the reproductive transition and before primary branch initiation, *brl1* mutant inflorescence primordia were larger than those of wild-type. White lines in **(D)** and **(E)** indicate how measurements of length and width were taken for the inflorescence primordia. Scale bars = 200μm.

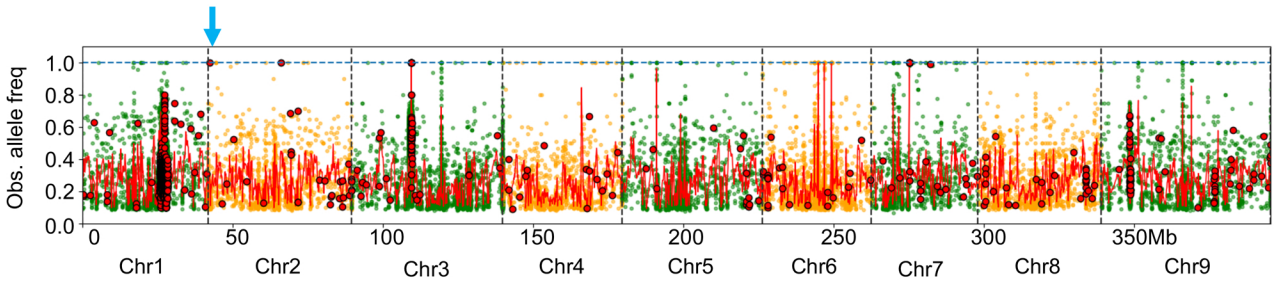

Supplemental Figure S2. BSA analysis of the *brl1* locus.
BSA was performed for the *brl1* mutant. Genomic position is plotted on the x axis and observed mutant allele frequency on the y axis. Green and orange dots represent all SNPs of the *brl1* mutant genomic pool compared to the A10.1 reference genome and red dots represent nonsynonymous SNPs. The red line is a smoothed curve over a 10 SNP window. **Blue arrow** indicates the location of the SNP that disrupted the start codon of *SvFul2.* The SNP locates at the beginning of chromosome 2 where the recombination rate are relatively high to break the linkage, which causes no clear peak around this region.

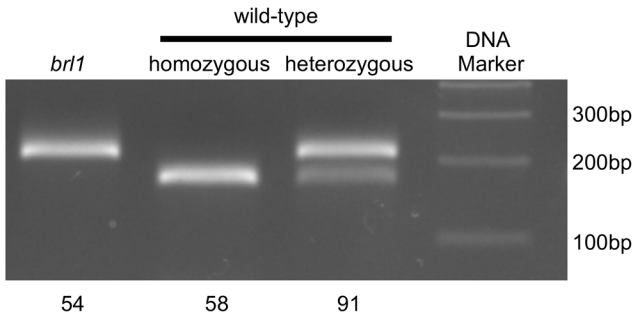

Supplemental Figure S3. Genotyping of *brl1* F_2 seedlings using a designed dCAPS marker for the SNP located at the start codon of *SvFul2*.

A mismatch was introduced using a primer to specifically bring a FokI cutting site in the wild-type allele for a dCAPS marker. The wild-type allele was sensitive to restriction enzyme digestion by FokI and produced a smaller band. The mutant allele was not digestible by the restriction enzyme and produced a larger band. Numbers beneath lanes indicate the number of individuals identified in each genotype.

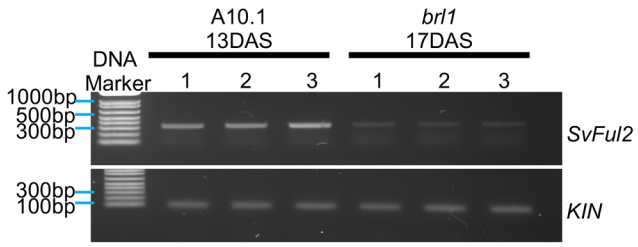

Supplemental Figure S4. RT-PCR results showing the reduced *SvFul2* expression level in mutant inflorescence primordia compared with A10.1.

Primers specific to *SvFul2* amplify a fragment of *SvFul2* transcript (**upper panel**) and primers specific to *KIN* amplify a fragment of transcript encoded KIN which was used as reference gene (**lower panel**). 1, 2, and 3 indicate biological replicates which contained a pool of inflorescence primordia. DAS: days after sowing.

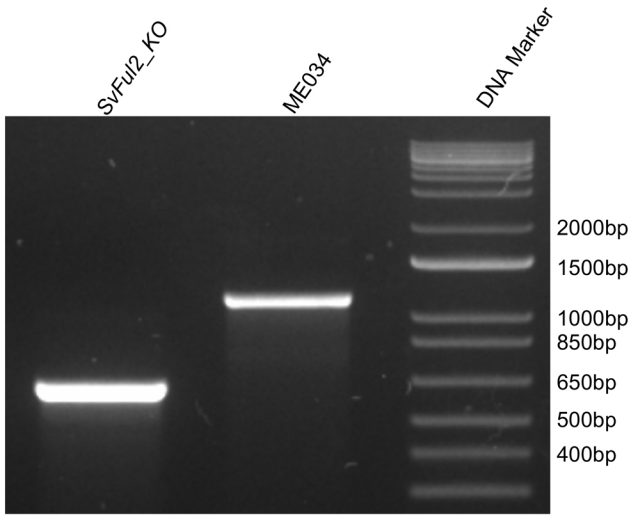

Supplemental Figure S5. PCR genotyping of the *SvFul2_KO* CRISPR edited line.
Primers flanking the deletion amplify the wild-type and CRISPR edited alleles.

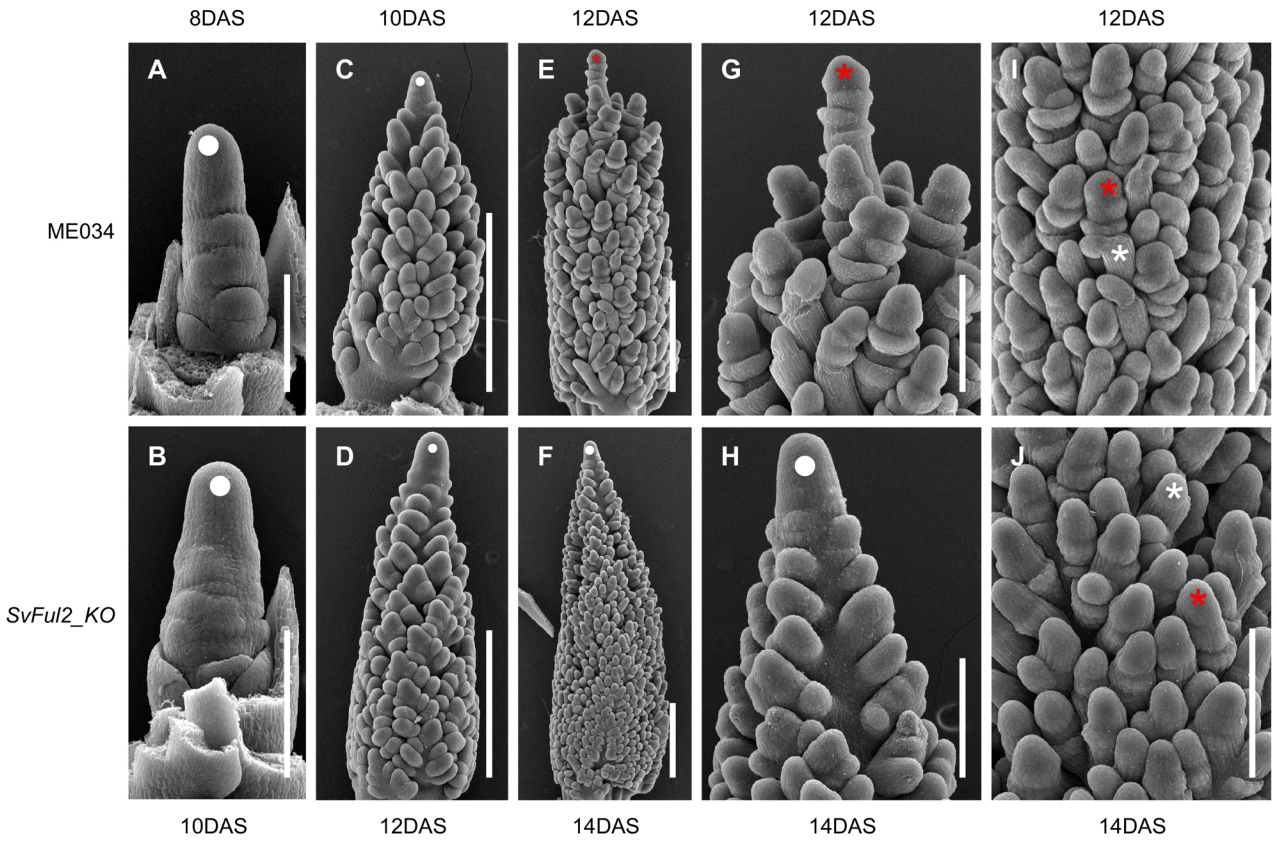

Supplemental Figure S6. Morphological analysis of early inflorescence development in *SvFul2_KO* by SEM.
The transition from SAM to IM (**white dot**) in *SvFul2_KO* plants was delayed to 10 DAS (**B**) compared to 8 DAS in ME034 (**A**).
Branching capacities were increased in *SvFul2_KO* panicles (**D** and **F,** 12 DAS and 14 DAS, respectively) compared to those of ME034 (**C** and **E**, 10 DAS and 12 DAS. respectively).
In ME034, the 12 DAS IM ceased to produce new BMs and terminated as the first SM (**G**, **red asterisk**) and then BMs started to differentiate into SMs (**I**, **red asterisk**) and bristles (**I**, **white asterisk**) basipetally. However, at 14 DAS, *SvFul2_KO* IM continued initiating primary branches at the inflorescence tip (**H, white dot**), even after BMs acquired SM (**J**, **red asterisk**) and bristle (**J**, **white asterisk**) identities.
Scale bars = 200μm in **A, B** and **G**-**J**. Scale bars = 500μm in **C-F**. DAS: days after sowing.
